# Interactions of anti-COVID-19 drug candidates with hepatic transporters may cause liver toxicity and affect pharmacokinetics

**DOI:** 10.1038/s41598-021-97160-3

**Published:** 2021-09-08

**Authors:** Csilla Ambrus, Éva Bakos, Balázs Sarkadi, Csilla Özvegy-Laczka, Ágnes Telbisz

**Affiliations:** 1 SOLVO Biotechnology, Irinyi József street 4-20, 1117 Budapest, Hungary; 2 Institute of Enzymology, ELKH Research Centre for Natural Sciences, Magyar Tudósok krt. 2, 1117 Budapest, Hungary; 3 Doctoral School of Molecular Medicine, Semmelweis University, Tűzoltó u. 37-47, 1094 Budapest, Hungary; 4 Department of Biophysics and Radiation Biology, Semmelweis University, Tűzoltó u. 37-47, 1094 Budapest, Hungary

**Keywords:** Cell biology, Drug discovery, Diseases

## Abstract

Transporters in the human liver play a major role in the clearance of endo- and xenobiotics. Apical (canalicular) transporters extrude compounds to the bile, while basolateral hepatocyte transporters promote the uptake of, or expel, various compounds from/into the venous blood stream. In the present work we have examined the in vitro interactions of some key repurposed drugs advocated to treat COVID-19 (lopinavir, ritonavir, ivermectin, remdesivir and favipiravir), with the key drug transporters of hepatocytes. These transporters included ABCB11/BSEP, ABCC2/MRP2, and SLC47A1/MATE1 in the canalicular membrane, as well as ABCC3/MRP3, ABCC4/MRP4, SLC22A1/OCT1, SLCO1B1/OATP1B1, SLCO1B3/OATP1B3, and SLC10A1/NTCP, residing in the basolateral membrane. Lopinavir and ritonavir in low micromolar concentrations inhibited BSEP and MATE1 exporters, as well as OATP1B1/1B3 uptake transporters. Ritonavir had a similar inhibitory pattern, also inhibiting OCT1. Remdesivir strongly inhibited MRP4, OATP1B1/1B3, MATE1 and OCT1. Favipiravir had no significant effect on any of these transporters. Since both general drug metabolism and drug-induced liver toxicity are strongly dependent on the functioning of these transporters, the various interactions reported here may have important clinical relevance in the drug treatment of this viral disease and the existing co-morbidities.

## Introduction

COVID-19 is a devastating new viral disease still showing an overall fatality rate of about 2–3%, with numerous patients requiring intensive care treatment. Several repurposed drugs have been proposed to have therapeutic effects against Sars-CoV-2 both in vitro and in vivo, and currently the most widely studied and applied such agents include ivermectin (IVE), ritonavir (RIT), lopinavir (LOP), favipiravir (FAV) and remdesivir (REM)^1–9^. IVE is a frequently used anti-parasitic drug, especially effective in various tropical diseases. While IVE has also been indicated to inhibit the cellular replication of the SARS-CoV-2 virus^1^, clinical trials showed no convincing antiviral efficacy in this disease^10,11^. LOP and RIT are efficient HIV protease inhibitors and were also shown to have in vitro efficacy against SARS-CoV-2 replication^6,12^. However, clinical studies performed until now do not support their anti-COVID-19 efficiency, neither when applied separately, nor in a combination, named Kaletra^5^. Currently none of these compounds are promoted to be used in COVID-19 outside selected clinical trials^13^. FAV and REM are prodrugs invented earlier as antivirals, inhibiting viral RNA-dependent RNA polymerase. Currently these two drugs have emergency use authorization for COVID-19 treatment in several countries, with still ongoing clinical studies. Remdesivir can be applied in hospitals only, whereas favipiravir can be prescribed at earlier disease stages for non-hospitalized patients as well. Currently the usefulness of these therapies is questionable, although slight beneficial effects are probable^14^.

**Figure 1:**
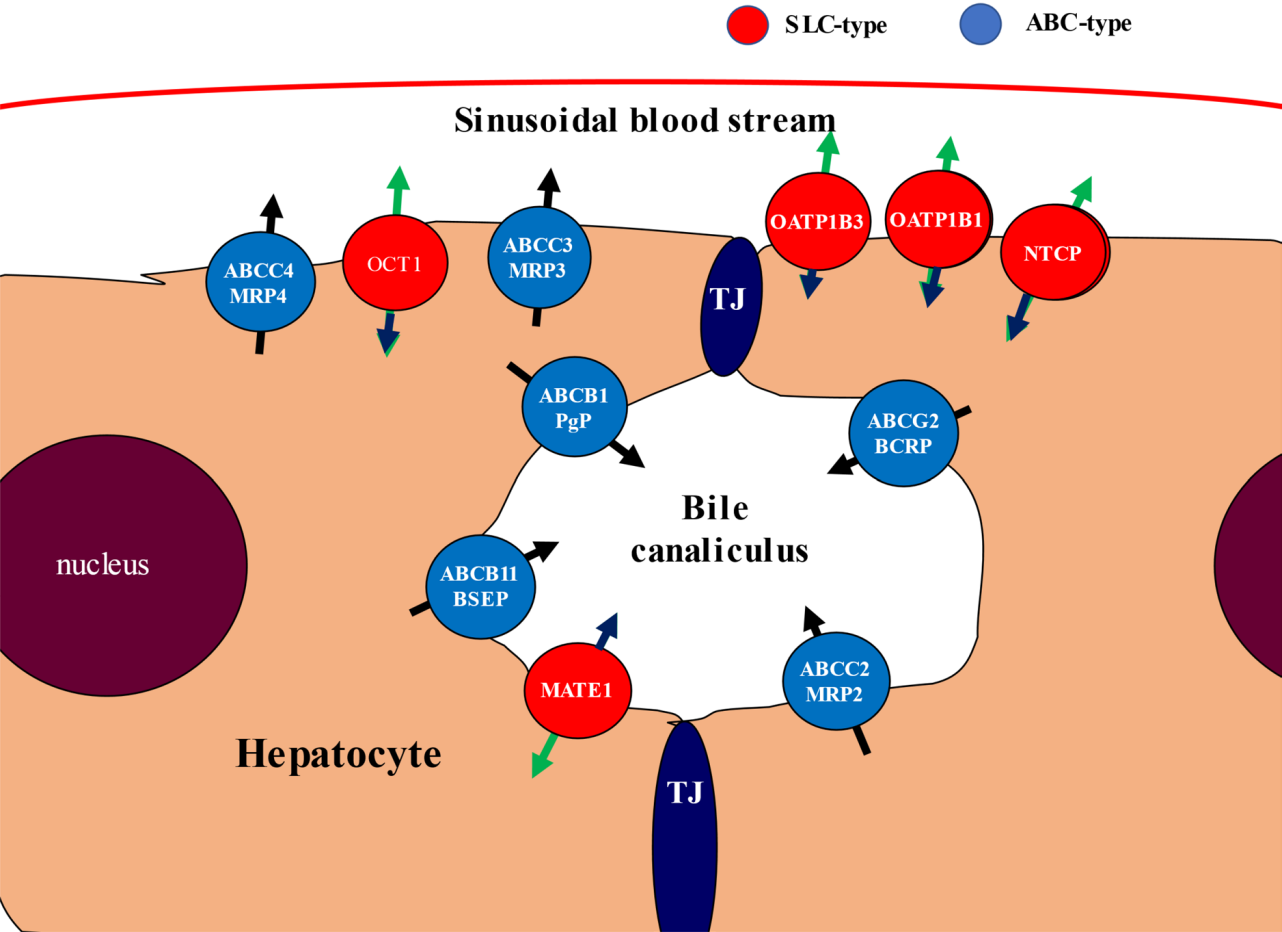
Schematic presentation of the localization and transport directions of the hepatocyte transporters examined in the present work. SLC type transporters are colored red, ABC type transporters are blue, TJ: tight junction.

Pharmacokinetic and pharmacodynamic characteristics, as well as toxic side effects of pharmaceutical agents are greatly influenced by membrane transporters, especially by those expressed in tissue barriers and in the central organ for drug metabolism, the liver (see Fig. 1). Canalicular ABC (ATP Binding Cassette) transporters in hepatocytes, such as ABCC2/MRP2, ABCG2/BCRP, ABCB1/MDR1/ P-gp and ABCB11/BSEP (bile salt export pump) mediate the extrusion of endo- and xenobiotics into the bile. P-gp, MRP2 and ABCG2 are multispecific transporters mediating the efflux of hydrophobic (P-gp) or partially detoxified amphiphilic compounds (MRP2, ABCG2). MRP2 is the key transporter for bilirubin conjugates. The SLC-type transporter MATE1 (SLC47A1—multidrug and toxin extrusion protein 1) in the hepatocyte canalicular membrane mainly transports cationic drugs, but also some zwitterionic and anionic molecules^15,16^, and mediates their biliary excretion. Inhibition of these drug exporters may cause elevated liver toxicity due to compound accumulation. ABCB11 (bile salt export pump, BSEP), the pump responsible for the extrusion of bile salts into the canaliculi, may also be inhibited by numerous drugs, leading to cholestasis or drug-induced liver injury (DILI)^17^.

Basolateral/sinusoidal ABC transporters (ABCC3/MRP3 and ABCC4/MRP4) defend the hepatocytes from any potential overaccumulation of toxic agents, including drugs, by their extrusion into the venous blood^18–23^. Key SLC-type (“uptake”) drug transporters from the sinusoidal blood stream include SLC22A1/OCT1**,** SLC10A1/NTCP, SLCO1B1/OATP1B1 and SLCO1B3/OATP1B3. OCT1 preferentially supports the hepatocellular entry of cationic agents, thus working in a coordinated fashion with the apical MATE1. NTCP primarily performs sodium-dependent uptake of bile acids in the basolateral membrane of hepatocytes, thus NTCP, together with BSEP, are major players in the enterohepatic circulation of bile acids. However, NTCP also promotes the hepatocyte uptake of several drugs, including statins, and therefore is a recognized risk factor for hepatic drug-drug interactions^24–26^. OATP1B1 and OATP1B3 are the key organic anion uptake transporters in the liver. Although with somewhat different selectivities and specificities, these transporters are responsible for the hepatocellular uptake and elimination of a wide variety of drugs and toxic compounds from the blood stream. Inhibition of these transporters causes an increase in blood retention and general toxicity of many clinically applied agents^27–30^.

While the transporter-related pharmacology and toxicology properties of LOP, RIT and IVE have already been examined in several studies, there are no detailed data available for REM and FAV. Previously we have studied the interactions of these repurposed anti-COVID-19 drugs with tissue barrier membrane transporters^31^, while in the current work, using various in vitro assays, we have examined their interactions with the key hepatocellular transporters involved in hepatic drug clearance. Based on the data obtained, we also provide assessment of in vivo pharmacokinetic properties and potential drug-drug interactions for these compounds.

## Results

### Interaction of potential anti-COVID-19 drugs with hepatic ABC transporters

**Figure 2:**
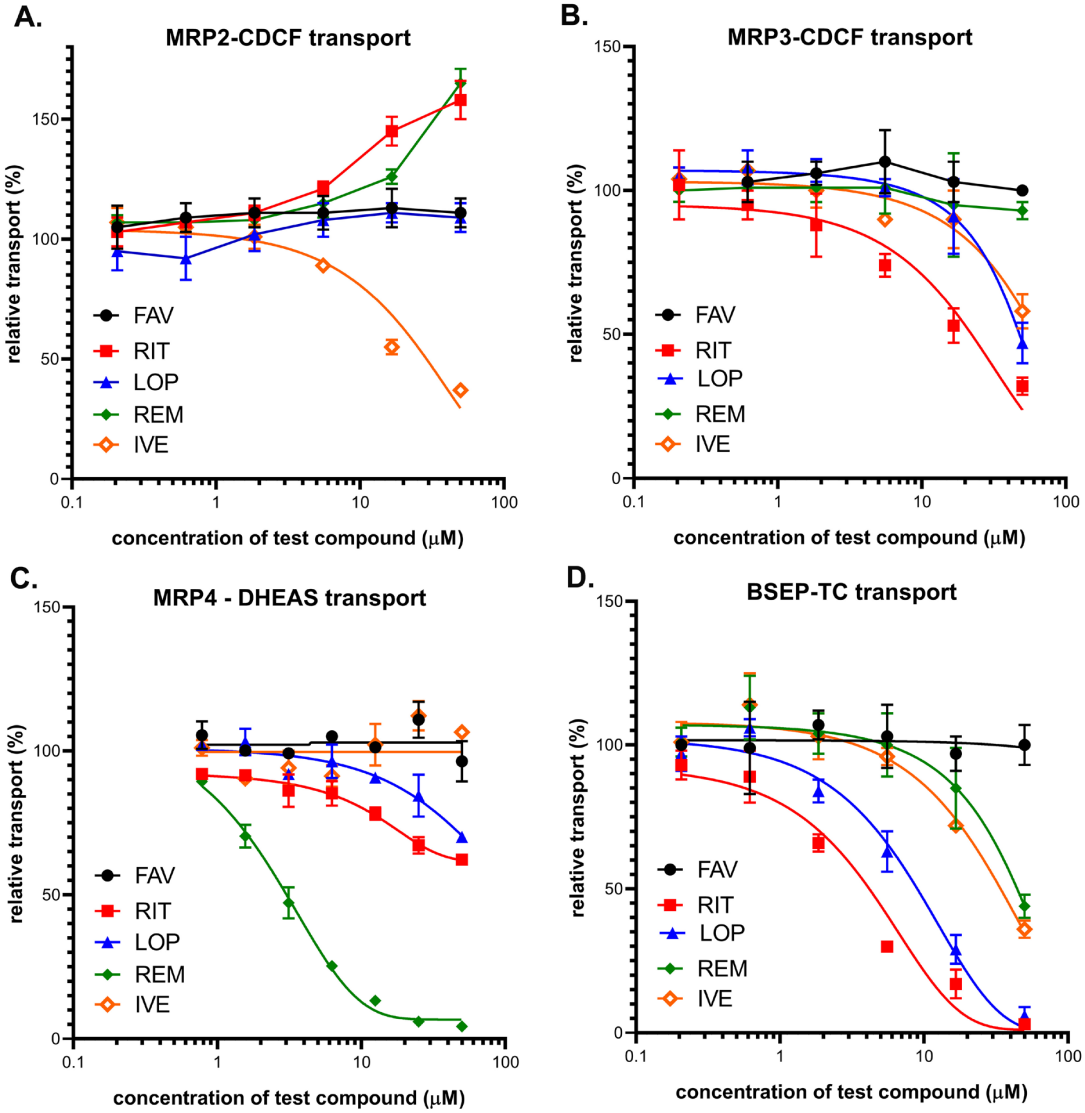
Inhibition of vesicular uptake of transporter-specific substrates by FAV, RIT, LOP, REM and IVE. (**A**) Inhibition of ATP-dependent vesicular uptake of CDCF in Sf9 cell membrane vesicles overexpressing MRP2. 30 µg (protein content) membrane vesicle was incubated with 5 µM CDCF at 37 °C for 10 min. Average values of 3 independent experiments +/− SE are shown. (**B**) Inhibition of ATP- and benzbromarone sensitive CDCF uptake into inverted HEK cell membrane vesicles overexpressing MRP3. 30 µg membrane vesicle was incubated with 5 µM CDCF at 37 °C for 10 min. Average values of 3 independent experiments +/− SE are shown. (**C**) Inhibition of ATP- dependent DHEAS uptake into inverted HEK cell membrane vesicles overexpressing MRP4. 50 µg membrane vesicle was incubated with 0.5 μM DHEAS at 32 °C for 1.5 min. Average values of 3 independent experiments +/− SE are shown. (**D**) Inhibition of ATP- and glyburide-sensitive taurocholate (TC) uptake into inverted HEK cell membrane vesicles, overexpressing BSEP. 50 µg membrane vesicle was incubated with 2 µM TC at 37 °C for 10 min. Average values of 3 independent experiments +/− SE are shown.

**MRP2** drug interactions were characterized by following the inhibitory effects of drugs on ATP- and benzbromarone-sensitive CDCF uptake into inverted membrane vesicles generated from MRP2-overexpressing Sf9 cells (Fig. 2A and Table 1). We found that IVE strongly decreased the accumulation of the probe substrate at higher than 10 µM concentration, while LOP and FAV had no such effect. Interestingly, RIT and REM did not inhibit but rather increased the probe substrate transport at high concentrations, possibly due to an allosteric effect^32,33^. Since reduced glutathione (GSH) levels may affect MRP2-dependent substrate transport or drug interactions, we have also examined these transport processes in the presence of 2 mM GSH, but neither the vesicular uptake of CDCF, nor its inhibition by the drugs examined were affected (data not shown).

**Table 1:** Summary of the transporter modulation properties of the repurposed anti-COVID-19 drugs examined in transport assays. Approximate IC_50_ (μM) values were determined by nonlinear regression analysis of the data shown in the results section, using GraphPad prism software (version 5.01, GraphPad, La Jolla, CA, USA). Favipiravir (not listed) had no effect on any of the transporters examined. Estimation of the DDI effects was performed as described in the Methods section, and references for in vivo parameters were provided in the Discussion. For importers the R values (cut off value 1.1), while for the exporters C_max_/IC_50_ values (cut off value 0.1) were calculated. For lopinavir and ritonavir R values were calculated for a combined treatment.

Table 1/A. Effects of drugs on hepatocyte exporters (basolateral: MRP3 and MRP4, apical: BSEP, MRP2 and MATE1)										
Hepatocyte exporters	ABCC3/MRP3		ABCC4/MRP4		ABCB11/BSEP		ABCC2/MRP2		MATE1/SLC47A1	
	Type	IC_50_ µM	Type	IC_50_ µM	Type	IC_50_ µM	Type	IC_50_ µM	Type	IC_50_ µM
LOP	inh	> 40	No effect	–	inh	7.8	No effect	–	inh	7.7
RIT	inh	17	No effect	–	inh	3.2	Increase	–	inh	1.4
IVE	inh	> 50	No effect	–	inh	≥ 30	inh	≥ 20	No effect	–
REM	No effect	–	inh	2.9	inh	≥ 40	Increase	–	inh	2.3

Table 1/B: Effects of drugs on hepatocyte importers (uptake transporters)										
Hepatocyte importers	OATP1B1			OATP1B3			NTCP/SLC10A1		OCT1/SLC22A1	
	Type	IC_50_ µM	R value	Type	IC_50_ µM	R value	Type	IC_50_ µM	Type	IC_50_ µM
LOP	inh	1.1	2.39	inh	2.6	1.74	inh	≥ 50	inh	> 50
RIT	inh	1.4		inh	1.5		inh	≥ 50	inh	5.9
IVE	inh	≥ 20		inh	1.4	1.07	no	–	no effect	–
REM	inh	2.9		inh	4.3		inh	≥ 50	inh	6.1

Table 1/C: Effects of drugs on the hepatocyte exporters ABCB1/Pgp and ABCG2/BCRP, as measured previously in similar vesicular transporter assay systems (Telbisz et al., 2021)										
Hepatocyte exporters	ABCB1/Pgp			ABCG2/BCRP						
	Type	IC_50_ µM	C_max_/IC_50_	type	IC_50_ µM	C_max_/IC_50_				
LOP	inh	0.6	18.6	inh	4.2	3.33				
RIT	inh	0.3	2.66	inh	7.5	0.13				
IVE	inh	0.3	0.17	inh	1.1	0.047				
REM	inh	> 20	n.r	inh	> 50	n.r				

**MRP3** drug interactions were characterized by measuring ATP- and benzbromarone sensitive CDCF uptake into inverted HEK-293 membrane vesicles generated from MRP3-overexpressing HEK-293 cells (Fig. 2B and Table 1). Only RIT inhibited the MRP3 transport function at a lower than 10 µM concentration, whereas LOP and IVE were effective only at higher concentrations (> 20 µM), while FAV and REM did not influence MRP3 transport at all.

**MRP4** drug interactions were characterized by measuring ATP-sensitive DHEAS (dehydroepiandrosterone-sulfate) uptake into inverted membrane vesicles generated from MRP4 overexpressing HEK-293 cells (Fig. 2C and Table 1). We found that most drugs examined had no significant inhibitory effect on MRP4-dependent substrate transport (a maximum of 10–30% inhibition was observed by up to 50 µM of RIT, LOP, IVE or FAV), while REM had a strong inhibitory action on this transporter, with an IC_50_ of about 2.3 µM (Fig. 2C).

**BSEP** drug interactions were characterized here by measuring the effects of drugs on ATP- and glyburide sensitive taurocholate (TC) uptake into inverted cell membrane vesicles generated from BSEP overexpressing HEK-293 cells (Fig. 2D and Table 1). We found that LOP and RIT strongly inhibited TC transport at low concentrations (see Table 1 for IC_50_ values) whereas IVE and REM were only effective above 20 µM. FAV did not modify BSEP-mediated TC transport.

### Cellular assays for SLC type transporters

**Figure 3:**
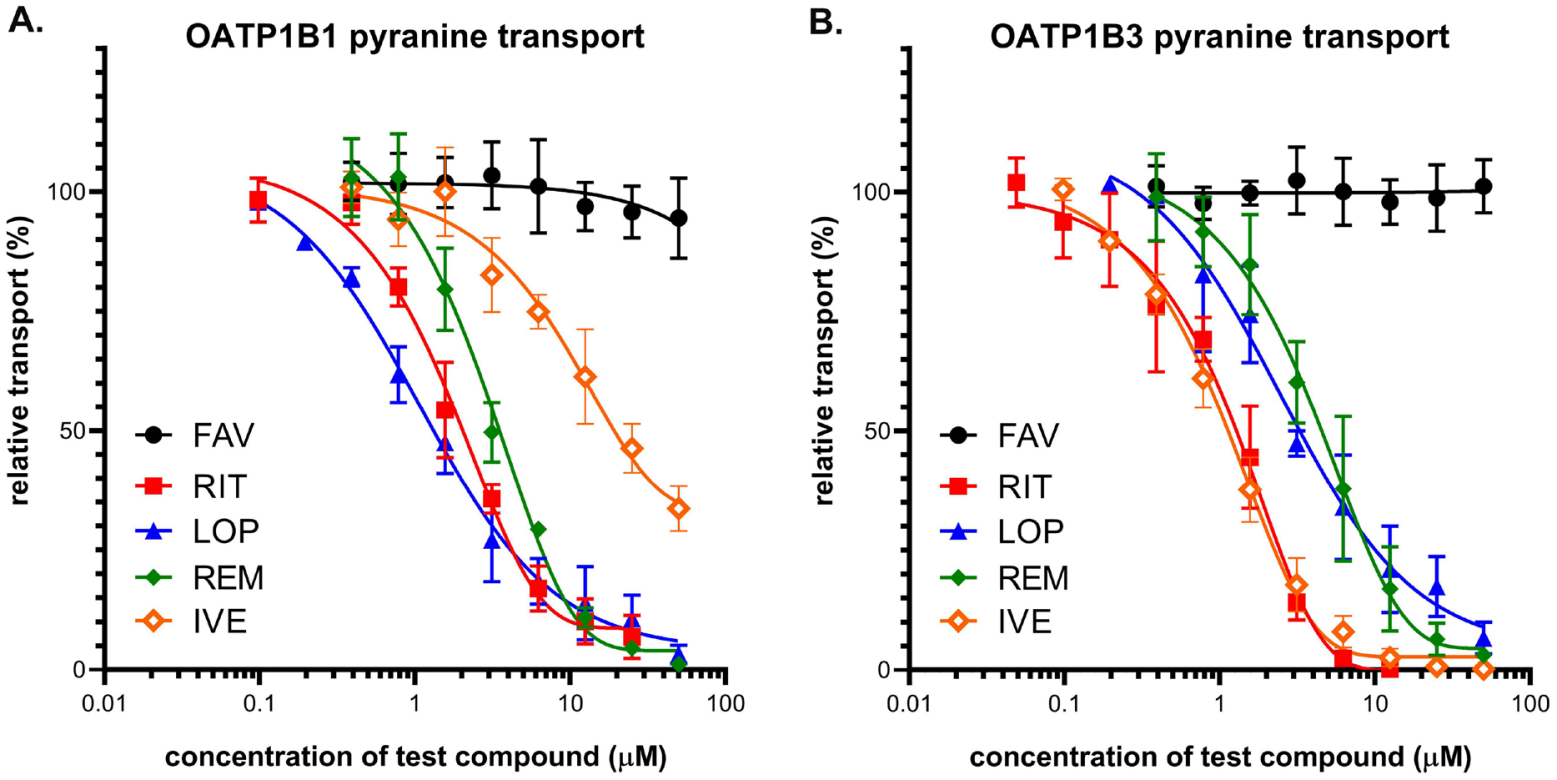
Inhibition of OATP1B1 (Panel **A**) and OATP1B3 (Panel **B**) dependent pyranine uptake in transporter overexpressing A431 cells by repurposed anti-COVID agents. Cells were incubated with 10 µM (OATP1B1) or 20 µM (OATP1B3) pyranine in the presence or absence of increasing concentrations of anti-COVID agents at 37 °C for 15 or 30 min (OATP1B1 or OATP1B3, respectively). Average values of 3 independent experiments +/− SD are shown.

**OATP1B1** (Fig. 3A) and **OATP1B3** (Fig. 3B) drug inhibition was studied by measuring the uptake of the fluorescent model substrate, pyranine, into A431 cells overexpressing the respective transporters and into mock-transfected control cells. As shown on Fig. 3, the drug inhibition panel for the two OATPs examined was somewhat different: IVE was less inhibitory for OATP1B1 than for OATP1B3, and RIT, LOP and REM had strong inhibitory effect at low micromolar range but with slightly different IC_50_ values for both transporters (see Table 1). FAV did not modify OATP functionality in our assay.

**Figure 4:**
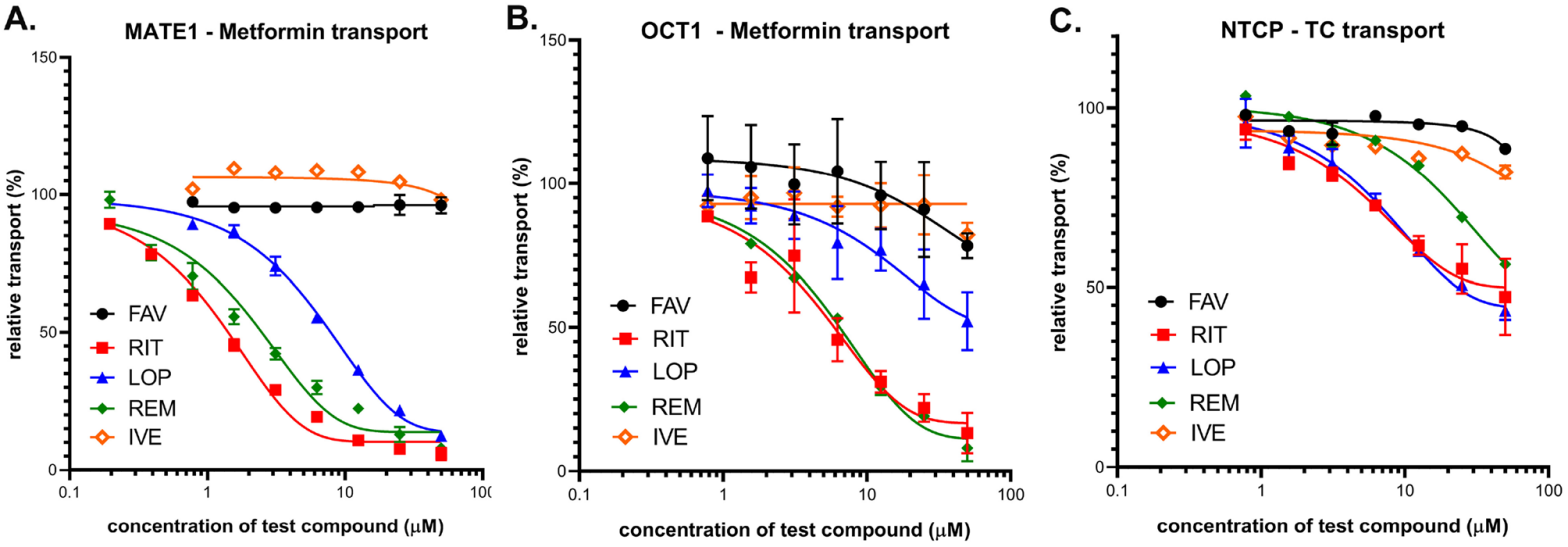
Inhibition of the cellular uptake of transporter-specific substrates by the repurposed anti-COVID agents FAV, RIT, LOP, REM and IVE. (**A**) Inhibition of MATE1 dependent metformin uptake in MATE1-overexpressing MDCKII cells. Uptake was measured by 10 µM metformin for 10 min at 37 °C. Average values of 3 independent experiments +/− SE are shown. (**B**) Inhibition of OCT1-dependent metformin uptake in OCT1-overexpressing HEK cells. Uptake was measured by 10 µM metformin for 5 min at 37 °C. Average values of 3 independent experiments +/− SE are shown. (**C**) Inhibition of NTCP-dependent taurocholate uptake in NTCP-overexpressing HEK cells. Uptake was measured by 2 µM taurocholate for 2 min at 37 °C. Average values of 3 independent experiments +/− SE are shown.

**MATE1 (SLC47A1)** interaction was characterized by metformin uptake into MDCKII cells overexpressing MATE1 and in mock-transfected controls. As shown in Fig. 4A, LOP, RIT and REM had a strong inhibitory action on this transporter at the low micromolar range, while IVE and FAV showed practically no inhibition of MATE1 transport activity (see also Table 1).

**OCT1 (SLC22A1)** interaction was measured by metformin uptake into HEK-293 cells overexpressing OCT1 and mock-transfected controls. As shown in Fig. 4B, RIT and REM had a strong inhibitory effect on this transporter (below 10 µM), while LOP was less inhibitory, and IVE and FAV had no significant inhibitory action on OCT1 transport (see also Table 1).

**NTCP/SLC10A1** interaction was investigated in HEK-293 cells overexpressing NTCP, where taurocholate (TC) uptake was measured in NTCP-overexpressing and in mock-transfected control cells. As shown in Fig. 4C, the repurposed anti-COVID agents had relatively weak inhibitory action on this uptake transporter: RIT, LOP and REM had IC_50_ values at or above 50 µM, and IVE and FAV showed only minor inhibition, and observed only at higher concentrations.

## Discussion

In the present work we have studied transporter interactions of several repurposed potential anti-COVID-19 agents. We focused on key membrane transporters involved in the liver uptake and excretion of endo- and xenobiotics and applied well documented in vitro assay techniques to characterize these interactions. We selected lopinavir, ritonavir, ivermectin, remdesivir and favipiravir as promising repurposed anti-COVID-19 agents for these studies. Some of the data presented here reinforces previously described transporter-drug interactions. In addition, our study on the key hepatocyte transporters working in a network at the uptake and export sites, may help decipher both potential risks of drug-induced liver injury (DILI) and alterations in general hepatocellular functions. Importantly, in the case of the clinically most promising agents, remdesivir and favipiravir, our findings may serve as a valuable addition to the previously scarce information about transporter interaction of these drugs.

In the case of ABC transporters, we employed a vesicular transport assay system based on inside-out vesicles generated of membrane isolates in which modulatory effects of the drugs on the accumulation of model substrates could be quantitatively examined. For SLC-type transporters we used whole-cell assays, in which drug modulation of a transporter specific substrate uptake was measured. In order to provide an estimation of potential in vivo drug effects from in vitro data, we followed the recommendations of the International Transporter Consortium (ITC)^34^ and used the available pharmacokinetic data for the given compounds, to indicate potential drug-drug interaction risks (DDI)^34^. We used the recommended in vivo*—*in vitro extrapolation approach, based on the R values for OATP transporters and the C_max_/IC_50_ values for ABC transporters (see the Methods section). In this extrapolation, our IC_50_ values and the FDA-published pharmacokinetic data for these drugs were used.

In harmony with earlier investigations, our results show that IVE has a strong in vitro inhibitory effect on ABCB1, ABCG2 and OATP1B3 transporters (^13,35–37^, Table 1). According to the FDA guideline for stromectol (IVE), we calculated the possible drug influence using a 165 µg/kg IVE dose and a peak plasma concentration value of 46 ng/ml (https://www.accessdata.fda.gov/drugsatfda_docs/label/2008/050742s022lbl.pdf). The predicted R value for IVE in the case of OATP1B3 (1.07) is near to the 1.1 cut-off value, and the C_max_/IC_50_ value is above the 0.1 cut-off value for ABCB1 (0.17) but not for ABCG2 (0.047). These data suggest that higher dose IVE treatment may increase the risk of DDI related to the interactions with these transporters. As widely accepted, mainly the ABCB1/P-gp and the ABCG2/BCRP efflux transporters are responsible for reduced human IVE toxicity, by restricting the absorption of this drug in the intestine and protecting the brain as part of the blood–brain barrier^35,36,38,39^. Based on our results, MRP2 and BSEP inhibition appears only at higher IVE concentrations (above 20 µM), which probably does not occur in vivo, but compromised ABCB1 and ABCG2 transporter function (mutations, inhibition) may increase available drug plasma levels.

LOP and IVE were suggested to be used in combination (Kaletra) for antiviral treatment^5^. It has been reported that these drugs strongly inhibited several drug transporters in a wide array of in vitro assays^17,37,40–45^, and here we have also found that LOP and RIT inhibited several hepatic transporters with high affinity (IC_50_-s below 10 µM, see^31^ and Table 1). According to an FDA dataset for Kaletra, the potential treatment dose is 400 mg lopinavir and 100 mg ritonavir, added in combination as an antiviral treatment (https://www.accessdata.fda.gov/drugsatfda_docs/label/2013/021251s046_021906s039lbl.pdf). This dose results in about 11–20 µM of lopinavir and 1–1.73 µM of ritonavir peak plasma concentrations in vivo^46^. The estimated R values for OATP1B1 (2.39, combined) and OATP1B3 (1.74, combined), and the C_max_/IC_50_ values for ABCB1 (18.6 for LOP, 2.66 for RIT) and ABCG2 (3.33 for LOP, 0.13 for RIT) indicate that LOP and RIT may exceed the cut-off values of these transporters and thus, inhibition of these transporters can be a relevant factor in DDI risk. Though the RIT dosage is relatively low in Kaletra, the measured low IC_50_ values indicate that RIT may exacerbate lopinavir-mediated transporter inhibition in the combination treatment.

Estimation of clinical relevance from the in vitro inhibition data regarding the other investigated transporters is more difficult. However, the low in vitro IC_50_ values (Table 1) for the OCT1 and the MATE1 transporters raise the possibility of potentially compromised in vivo function of these transporters. Previously, RIT was classified as a highly potential DILI causing compound, based on information in the NIH LiverTox Database^47^ (livertox.nih.gov) and the Liver Toxicity Knowledge Base (fda.gov), although the cause of the observed liver enzyme elevations during ritonavir therapy has not yet been fully understood. The risk of DILI by RIT and LOP may occur via several mechanisms. It is known that these drugs are extensively metabolized via CYP3A4, and the production of toxic intermediates as well as their interactions with multidrug transporters may be involved in liver toxicity. Another significant source of DILI can be a compromised bile acid handling. If hepatocellular concentrations of these drugs reach the micromolar range, the bile acid exporter activity of BSEP may be decreased, because the IC_50_ values of LOP and RIT regarding BSEP were 7.8 and 3.2 µM, respectively. Indeed, even low potency BSEP inhibition may be biologically significant if the local drug exposure is high. It has been reported that basolateral MRP3 and MRP4 provide a compensatory mechanism for bile acid efflux in the case of BSEP inhibition^48^ and, although these transporters also interact with RIT and LOP, the high IC_50_ values indicate that RIT and LOP may not inhibit the function of these transporters in vivo. However, the combined inhibition of BSEP and these transporters may lead to a clinically significant DILI, especially in the case of a wide-spread inflammation caused by a Sars-CoV-2 infection. In addition, pharmacokinetics of other drugs, optionally co-dosed in COVID patients, may be influenced by LOP/RIT. For example the transport of OATP1B substrate drugs, including statins, repaglinide, olmesartan and valsartan^28,49,50^, or the OCT1- and MATE1-mediated metformin transport^51^ could also potentially be affected by LOP and/or RIT. Consistently with in vitro findings of transporter inhibition studies, Macias et al*.* found in a cross-sectional study a high overall frequency of DDI between ritonavir-boosted lopinavir for treating COVID-19 and other relevant medications such as statins, antibiotics, corticosteroids, or antiarrhythmic drugs^52^.

Since REM is a most promising repurposed drug in treating COVID-19, in this work we carefully assessed potential transporter inhibitions by this compound in various assay systems. REM did not show a significant inhibition of the ABC transporters, while we observed strong inhibition of OATP transporters (IC_50_ of OATP1B1 and 1B3 were 2.9 and 4.3 µM, respectively) (Table 1). Moreover, OCT1- and MATE1-mediated in vitro metformin transport was inhibited by REM in a concentration-dependent manner with an IC_50_ of 6.1 and 2.3 µM. These in vitro IC_50_ concentrations are close to the peak plasma concentration observed 30 min after intravenous administration of 200 mg REM for 30 min^53^, or close to the 3.7 µM C_max_ after intravenous administration of a clinically applied dose of 100 mg remdesivir in healthy adults (https://www.accessdata.fda.gov/drugsatfda_docs/label/2020/214787Orig1s000lbl.pdf).

Based on our data, a combined inhibition of these transporters may affect the uptake and the biliary extrusion of both anionic and cationic drugs as well as endogenous substates, such as bile acids. REM also inhibited the NTCP-mediated uptake of taurocholate in a concentration-dependent manner. As NTCP and BSEP inhibition in vitro occurred only at high REM concentrations (40–50% inhibition at 50 µM) probably not achieved in vivo, this effect may not be considered clinically relevant. REM was shown to inhibit MRP4 in vitro (IC_50_ was 2.9 µM), whereas it did not inhibit either MRP2 or MRP3. Since MRP4 has the ability to transport nucleoside analogue drugs^21,54^, this relatively selective effect was not unexpected. Regarding MRP4, there are no specific citations of clinically relevant DDIs ascribed to this transporter, and there is only limited information on the role of MRP4 in the clinical ADME of drugs. However, MRP4 is a multispecific pump extruding endogenous metabolites, including bile acids, urate, and conjugated steroid hormones into the bile canaliculi, therefore its potential involvement in adverse effects and DDI cannot be excluded. When treating co-morbidities in COVID-19, clinically important MRP4 drug substrates include cephalosporin, several antibiotics, diuretics like furosemide and hydrochlorothiazide, as well as olmesartan^18,21–23,54–58^. In addition, MRP4 inhibitors include non-steroidal anti-inflammatory and cardiovascular drugs^54^. It should be noted that while in this work we focused on liver resident transporters, MATE1 and MRP4 are also highly expressed in the basolateral renal tubular membranes and these proteins have an important role in renal drug excretion. The current FDA guideline recommends evaluation of MATE1-mediated interactions for drugs that undergo significant renal secretion (≥ 25% of total systemic clearance). Thus, REM inhibition of MRP4 may have multiple effects on general uric acid and drug clearance. In addition, MRP4 in the intestinal epithelia is involved in the oral absorption of various drugs, while in the blood–brain barrier MRP4 has a protective role for the central nervous system from drugs^20,57^.

In our REM studies, the data support earlier observations (https://www.ema.europa.eu/en/documents/other/summary-compassionate-use-remdesivir-gilead_en.pdf) on the inhibition of OATP1B1, OATP1B3, BSEP, MRP4, and NTCP, while MATE1 inhibition is a new information. Since REM is rapidly cleared (T1/2 = 69 min)^53^ from the human body, the potential of REM to be a perpetrator of clinically significant drug-drug interactions through these hepatic transporters is limited.

As a summary, we found that the examined repurposed anti-COVID-19 agents, with the exception of FAV, have multiple inhibitory effects on hepatic membrane transporters. These results may significantly help to decipher the effects of these transporter-drug interactions on general drug clearance and drug-induced liver toxicity. In addition, the various interactions quantitatively assessed here should be considered in clinical drug treatment strategies for the COVID-19 disease and for understanding existing co-morbidities.

## Methods

**The source of materials** is provided in the detailed descriptions of the methods applied. Lopinavir, ritonavir and ivermectin were obtained from Sigma Aldrich Inc.; favipiravir (T-705) from MedChem Express; remdesivir was kindly provided by Lajos Szente, Cyclolab Ltd, Budapest, Hungary.

For the ***ABC-type transporter assays*** we used isolated membrane vesicle-based functional assays. ABCC2/MRP2 vesicles were prepared from Sf9 cells infected by the human MRP2 coding baculovirus using a previously established method^33^. ABCB11/BSEP, ABCC3/MRP3 and ABCC4/MRP4 membranes were prepared from transporter expressing HEK-293 cells by Solvo Biotechnology (Budapest, Hungary). The vesicular uptake of transporter specific substrates was measured, and transporter related uptake was defined as the difference between the uptake with and without ATP (in the presence of AMP). Transporter specific inhibitors were also applied to control the specificity of transporters. For BSEP, radiolabeled taurocholate (1.5 µCi/sample of radiolabeled compound plus 2 µM unlabeled TC) (PerkinElmer Co); for MRP2 and MRP3, CDCF (5(6)-Carboxy-2′,7′-dichlorofluorescein, Sigma) (5 µM); for MRP4, radiolabeled dehydroepiandrosterone sulfate (DHEAS) (0.2 µCi radiolabeled compound plus 0.5 µM unlabeled DHEAS) (PerkinElmer Co) substrates were used. Applied specific inhibitors were 100 µM glyburide for BSEP, 40 µM benzbromarone for MRP2 and MRP3, and 50 µM MK571 for MRP4. A 30 µg (MRP2, MRP3) or 50 µg (BSEP, MRP4) protein/sample was incubated for 10 or for 1.5 min (MRP4) with the specific substrate at 37 °C or at 32 °C (MRP4) in the presence of test drugs (added in 1 µl of DMSO) and incubation was started by pipetting 4 mM Mg-ATP or Mg-AMP to the samples. DMSO was added to the controls as well. The sample volume was 75 µl. After the incubation, samples were filtrated and washed rapidly on the Millipore manifold vacuum filter in MSFBN6B10 (Millipore, Burlington, MA, US) filter plate. After the plates were dried, Optiphase HiSafe (PerkinElmer) scintillation cocktail was added to each well for detection of radiolabeled compounds. CDCF was dissolved from the filter by 10% SDS and as a fluorescence stabilizer 0.1 N NaOH was added. The amount of radiolabeled substrates inside the filtered vesicles was determined by liquid scintillation counting by a MicroBeta Scintillation Counter (PerkinElmer, Waltham, MA, US). CDCF fluorescence was measured by VictorX (PerkinElmer Perkin-Elmer, Waltham, MA, US) plate reader at 492/635 nm.

For the ***SLC type transporter assays*** we used whole cell assays, by employing cell lines specifically expressing the investigated hepatic transporters and the relevant control, mock-transfected cell lines^59^.

For the ***OATP1B1 and OATP1B3 transporter assays,*** the interaction between potential anti-COVID-19 agents and OATPs was investigated in a microplate-based indirect assay employing pyranine as described^60^. Briefly, A431 cells (ATCC) overexpressing OATPs^60^ and mock transfected A431 cells as control were seeded on 96-well plates in a density of 8 × 10^4^ cells/well and cultured for 24 h at 37 °C. The next day, cell culture media was removed and the cells were preincubated in an uptake buffer in the presence or absence of anti-COVID-19 agents for 5 min at 37 °C. The reaction was started by the addition of an uptake buffer containing a final concentration of 10 μM (OATP1B1) or 20 μM (OATP1B3) pyranine and cells were incubated at 37 °C for 15 min (OATP1B1) or 30 min (OATP1B3). The reaction was stopped by removing the supernatant and the cells were washed with ice-cold PBS. The fluorescence of cells was determined using an Enspire fluorescent plate reader (PerkinElmer Co), Ex/Em: 460/510 nm. OATP-dependent transport was determined by extracting fluorescence measured in mock transfected cells.

***MATE1 (SLC47A1), OCT1 (SLC22A1) and NTCP (SLC10A1) transporter inhibition assays*** were performed as follows: overexpressing cell lines created by lentiviral method were used for these assays described in^61^. MATE1 was overexpressed in Madin-Darby canine kidney strain II (MDCKII) cells while OCT1 and NTCP were overexpressed in HEK-293 cells. Cells were seeded into 96-well tissue culture plates at a cell density of 1 × 10^5^ cells per well. Experiments were performed 16–24 h after seeding. Before the experiments, cells were pre-incubated at 37 °C for 5 min in a Krebs Henseleit buffer (KHB, pH 8.0) or Hank’s balanced salt solution (HBSS, pH 7.4) containing the tested drug at increasing concentration. DMSO was used for solvent control and the reference inhibitor (RI) for reference inhibitor wells. After the pre-incubation step, the solutions were removed. Experiments were performed at 37 °C in KHB or in HBSS containing the appropriate probe substrate and the drug at increasing concentrations. The organic solvent concentrations were equal in all wells. Cells overexpressing MATE1 and OCT1 were incubated with metformin (10 µM, including 0.2 or 0.1 μCi/mL ^14^C-metformin) for 10 or 5 min, respectively, while cells overexpressing NTCP were incubated with TC (2 µM, including 2 µCi/mL ^3^H-TC) for 2 min at 37 °C. MDCKII or HEK293-Mock (empty vector transduced) cells were used as a control. After the experiment, cells were washed twice with 100 µL of cold HBSS and lysed with 50 µL of 0.1 M NaOH and then analyzed by liquid scintillation.

### Data and statistical analysis

All experiments were performed in duplicates and repeated in three biological replicates, thus each data point reflects six independent measurements. The group sizes were equal in all experiments. Normalization for the per cent (%) of control values was performed after subtracting the background levels as specified in the assay descriptions above, in order to avoid the inherent differences of the transporter activities and to properly reflect the inhibitory potential of each drug examined. IC_50_ values were calculated by nonlinear regression analysis using the GraphPad prism software (version 5.01, GraphPad, La Jolla, CA, US https://www.graphpad.com/scientific-software/prism/).

For the estimation of potential in vivo drug effects, we have used the R values and the C_max_/IC_50_ values, calculated as described in the International Transporterter Consortium guideline^34^, and the available pharmacokinetic data for the compounds. The R value is defined as 1 + (fu × Iin, max/IC_50_), in which Iin, max is the estimated maximum inhibitor concentration at the inlet to the liver and is equal to: Iin, max = Cmax + (ka × Dose × FaFg)/Qh/RB, where C_max_ is the maximum systemic plasma concentration of the inhibitor; ka is the absorption rate constant. ka = 0.1/min was used as the worst-case estimate; Fa is the fraction absorbed, and Fa = 1 was used as the worst-case estimate. Fg is the intestinal availability, and Fg = 1 was used as the worst-case estimate; Qh is the hepatic blood flow rate. Qh = 1,500 ml/min was used; RB is the blood-to-plasma concentration ratio, assumed to be 1. IC_50_ is the drug concentration which caused in vitro measured half maximum inhibition of the given transporter.

## Data Availability

The datasets generated and/or analyzed during the current study are available from the corresponding author upon reasonable request.
